# Trait mindfulness, psychological skills, and mental toughness in paddleboard athletes: A correlational study

**DOI:** 10.1371/journal.pone.0354427

**Published:** 2026-09-10

**Authors:** Jiarong Wang, Roxana Dev Omar Dev, Nurhidayah Yaakop

**Affiliations:** 1 Department of Sport Studies, Faculty of Educational Studies, Universiti Putra Malaysia, Serdang, Selangor, Malaysia; 2 Faculty of Physical Education and Health, Sanming University, Sanming, Fujian, China; Shahrood University of Technology, IRAN, ISLAMIC REPUBLIC OF

## Abstract

A cross-sectional study was conducted with 172 athletes. Participants completed the Mindful Attention Awareness Scale (MAAS), the Athletic Coping Skills Inventory-28 (ACSI-28), and the Sport Mental Toughness Questionnaire (SMTQ). Results indicated moderate-to-high levels across all study variables, with no significant gender differences observed. Trait mindfulness was positively associated with psychological regulation processes (r = 0.642), and both variables were positively associated with mental performance expression (r = 0.498). Psychological regulation processes were strongly associated with mental performance expression (r = 0.772). A significant indirect association was observed between trait mindfulness and mental performance expression via psychological regulation processes (b = 4.197), based on bootstrap mediation analysis. These findings suggest that psychological variables in paddleboarding are characterized by a pattern of interrelated statistical associations among trait mindfulness, regulatory processes, and performance-related outcomes. The results contribute to sport psychology literature by clarifying the relational structure among these constructs in a balance-demanding sport context.

## Introduction

As competitive sports evolve toward higher performance demands, success depends not only on physical and technical factors but also on psychological processes involved in performance regulation [1]. Trait mindfulness refers to a dispositional tendency to attend to present-moment experiences with awareness and has been associated with attentional control and emotional regulation [2]. Psychological regulation processes (previously conceptualized as psychological skills), including attentional focus, coping with adversity, goal setting, and pressure regulation, represent learned strategies for managing competitive demands [3]. Mental performance expression (previously conceptualized as mental toughness), including confidence, constancy, and control, reflects athletes’ability to maintain performance under pressure [4]. Previous research has suggested associations among these variables in sport contexts [5].

Paddleboarding is a water-based sport characterized by continuous balance control and adaptation to variable environmental conditions [6]. Athletes must maintain postural stability while responding to wind, waves, and fatigue [7]. These demands require attentional regulation and coping responses during performance [8].

Although psychological factors have been widely studied in team, endurance, and individual sports, fewer studies have examined these variables in balance-based water sports such as paddleboarding [9]. Existing research has mainly focused on physiological and biomechanical factors [10], with limited attention to psychological variables [11]. This highlights a need to further examine psychological characteristics in this specific sport context.

Previous theoretical work has distinguished psychological demands, regulatory processes, and performance outcomes in sport contexts [12]. Based on this perspective, trait mindfulness may be associated with psychological regulation processes, which may also be associated with mental performance expression [13]. This study examines relationships among trait mindfulness, psychological regulation processes, and mental performance expression in competitive paddleboard athletes.

Within this theoretical framework, existing studies have predominantly examined mindfulness, psychological regulation processes, and performance outcomes in isolation or within linear associative models. However, less attention has been given to integrating these constructs within a unified self-regulation system in sport contexts characterized by high instability and continuous attention demands. It remains unclear whether the proposed relationships among trait mindfulness, psychological regulation processes, and mental performance expression can be interpreted as part of a coherent regulatory structure under sport-specific performance constraints, such as those observed in balance-based aquatic sports.

Previous research suggests that mindfulness-related processes may be associated with attentional and emotional regulation, which are in turn related to performance outcomes in sport [14]. Psychological regulation processes and mental performance expression are therefore treated as statistically associated variables rather than independent psychological constructs. Recent studies suggest that mindfulness-based approaches may be relevant to psychological functioning in athletic populations [15].

The present study therefore aimed to examine relationships among trait mindfulness, psychological regulation processes, and mental performance expression in competitive paddleboard athletes [16], to test potential gender differences, and to examine whether psychological regulation processes are statistically associated with the relationship between trait mindfulness and mental performance expression.

### Conceptual model and hypotheses

The present study is grounded in a psychological self-regulation perspective in sport contexts, which conceptualizes psychological functioning as a system of interrelated regulatory processes that support goal-directed behavior. Within this framework, athletic performance is not determined by isolated psychological constructs, but by the coordinated functioning of attentional, emotional, and behavioral regulation systems. Self-regulation theory provides the primary explanatory lens for all hypothesized relationships in the present study.

Trait mindfulness is conceptualized as a dispositional factor associated with enhanced self-regulatory awareness, supporting the monitoring of internal states and external performance demands [17]. Psychological regulation processes (operationalized as psychological skills) represent functional processes within a self-regulatory system through which athletes manage attentional focus, emotional responses, and coping strategies under competitive stress [18].

Mental performance expression (operationalized as mental toughness) reflects a performance-related manifestation within the self-regulatory system, characterized by confidence, constancy, and perceived control during performance situations [19].

Rather than assuming a strict causal sequence, the present study adopts a statistical self-regulation model, in which trait mindfulness, psychological regulation processes, and mental performance expression are treated as interrelated components within a shared regulatory system [20].

**Fig 1:**
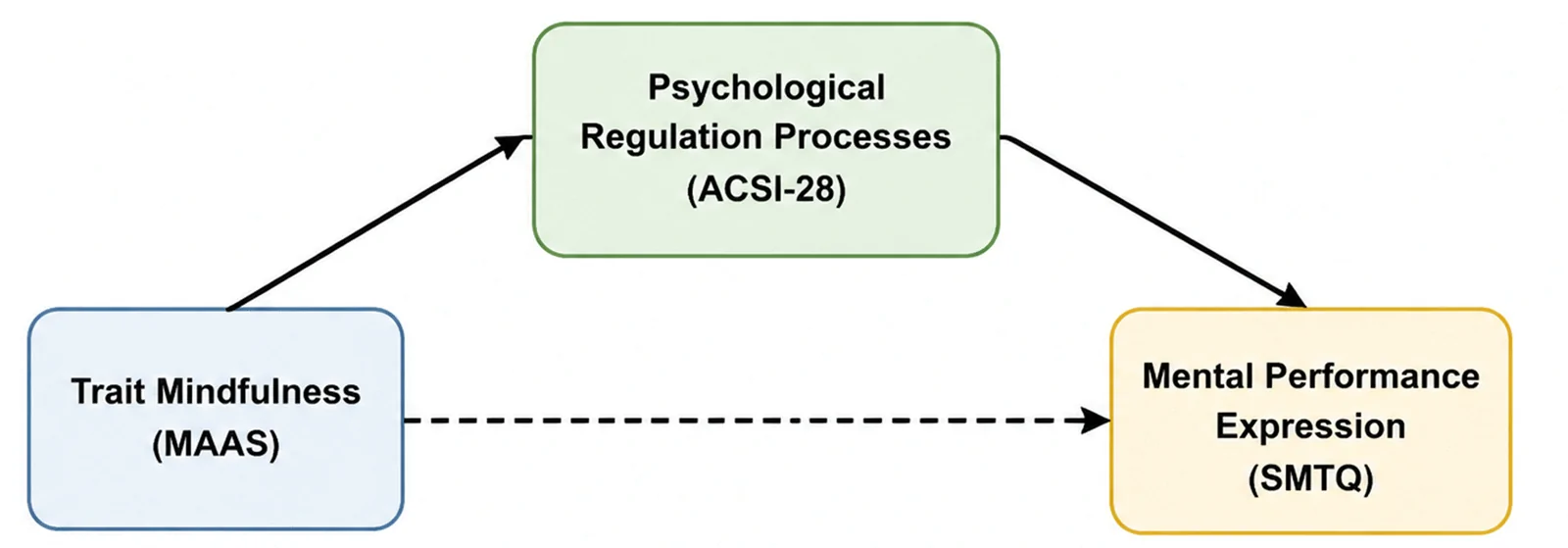
Statistical description of associations among sport psychological variables. **Note.** Trait mindfulness is conceptualized as a dispositional variable associated with sport psychological variables. Psychological regulation processes (ACSI-28) and mental performance expression (SMTQ) represent observed variables in this study rather than distinct latent constructs. Solid arrows indicate statistical associations (H1–H3). The dashed arrow indicates a statistical indirect association in cross-sectional data (H4). The figure is simplified for descriptive clarity.

Psychological regulation processes are examined as a statistical bridging construct linking mindfulness-related awareness and performance-related outcomes Fig 1.

Accordingly, the following research questions and hypotheses were formulated:

RQ1: What are the levels of trait mindfulness, psychological regulation processes, and mental performance expression among competitive paddleboard athletes?
RQ2: Are there gender differences in trait mindfulness, psychological regulation processes, and mental performance expression among competitive paddleboard athletes?
H1: Trait mindfulness is positively associated with psychological regulation processes among competitive paddleboard athletes.
H2: Trait mindfulness is positively associated with mental performance expression among competitive paddleboard athletes.
H3: Psychological regulation processes are positively associated with mental performance expression among competitive paddleboard athletes.
H4: Psychological regulation processes are examined as a statistical construct associated with trait mindfulness and mental performance expression in a cross-sectional covariance framework.

## Literature review

### Trait mindfulness and psychological skills in sport

Trait mindfulness is defined as a stable tendency to maintain awareness of present-moment experiences with reduced automatic processing [21]. Within sport settings, athletes with higher mindfulness levels tend to show superior attentional allocation, more effective emotional regulation [22], and reduced cognitive interference during performance situations. These characteristics are linked to enhanced use of psychological regulation processes (formerly psychological skills), such as maintaining attentional focus, managing pressure situations, and applying coping strategies during performance demands.

Mental toughness has been conceptualized as a multidimensional construct reflecting psychological resilience, consistency, and sustained performance capacity under pressure [23]. Within this framework, psychological regulation processes have been identified as key antecedents of mental performance expression (formerly mental toughness), which reflects stable patterns of confidence, persistence, and perceived control in competitive environments [24]. Empirical evidence suggests that athletes demonstrating stronger regulatory capacities tend to exhibit greater consistency in performance execution and improved responses to competitive setbacks [25].

In aquatic balance-based sports such as paddleboarding, these psychological components are particularly relevant due to unstable environmental inputs and continuous postural adjustment requirements [26]. Performance success in such contexts depends heavily on maintaining attentional stability and adaptive coping responses under fluctuating external conditions.

### Theory-driven integration

The relationships among trait mindfulness, psychological regulation processes, and mental performance expression are conceptualized in this study within a self-regulation framework in sport psychology [27,28]. Within this framework, psychological functioning is understood as a dynamic system of attentional, emotional, and behavioral regulation processes that jointly contribute to performance outcomes rather than isolated psychological traits.

Mental toughness has been extensively conceptualized as a multidimensional construct reflecting psychological resilience and performance stability under pressure [29], and is understood as a dynamic outcome of regulatory adaptation processes rather than a fixed trait-like characteristic [30].

Trait mindfulness is defined as a stable tendency to maintain present-moment awareness with reduced automatic cognitive processing [31]. In sport contexts, mindfulness is associated with improved attentional allocation and emotional regulation and is proposed to enhance metacognitive awareness and reduce automatic cognitive reactivity, thereby supporting more effective psychological regulation processes.

Psychological regulation processes (operationalized as psychological skills) represent functional mechanisms for managing attentional focus, emotional responses, and coping under competitive stress [32]. These processes are considered central pathways through which mindfulness-related cognitive states are translated into performance-relevant behaviors.

Mental performance expression (operationalized as mental toughness) reflects the behavioral manifestation of sustained self-regulation, including confidence, constancy, and perceived control under pressure [33].

Accordingly, trait mindfulness is proposed to support psychological regulation processes, which are associated with mental performance expression. This reflects a statistical self-regulation structure rather than a strict causal mediation model, in which psychological regulation processes serve as the central linkage between mindfulness and performance outcomes.

This conceptualization is consistent with prior evidence suggesting that mindfulness-based processes can influence psychological regulation and performance-related outcomes in athletes [34], and that psychological regulation processes may partially explain statistical associations between mindfulness and performance [35].

## Materials and methods

### Study design and research site

This study employed a cross-sectional design [36] to examine the relationships among trait mindfulness, psychological regulation processes, and mental performance expression in competitive paddleboard athletes. The study was conducted at the National Paddleboard Training Base of the University Sports Science Center, which provides standardized open-water training conditions, including calm-water and slow-wave practice areas, an internationally certified paddleboard coaching team, and a fixed competitive training cycle. Data was collected during scheduled testing sessions arranged around the athletes’ regular training program. All questionnaires were administered face-to-face by trained researchers to ensure standardized instructions and consistent data collection procedures.

### Ethical approval and informed consent

This cross-sectional study was approved by the Ethics Committee of the Faculty of Physical Education and Health, Sanming University (SMU-2025-SP-017). Written informed consent was obtained from all participants prior to participation. All participants were informed of the study purpose, procedures, voluntary nature of participation, and confidentiality of their responses. All procedures were conducted in accordance with institutional ethical standards and the Declaration of Helsinki [37].

### Participants and sampling procedure

Participants were recruited using cluster sampling from the University Competitive Paddleboard Professional Training Program. A total of 182 athletes were approached between 20 July and 19 August 2025. Inclusion criteria were: (a) at least one year of systematic paddleboard training; (b) participation in regional or national competitions within the past year; and (c) training at least three times per week. Additional inclusion criteria included age between 18 and 30, no diagnosed mental illness, and absence of musculoskeletal injury affecting posture or stability. Participants experiencing acute fatigue, illness, or training disruption on the testing day were excluded [38]. After screening, 172 valid questionnaires were retained (valid response rate 94.6%). Demographic data collected included gender, age, educational background, athletic level, daily training duration, paddleboard event, general health, and total years of paddleboard training.

All analyses were conducted within a cross-sectional covariance framework, and no causal interpretations were made regarding relationships among variables.

## Measures

### Conceptual alignment of constructs

Psychological skills and mental toughness are used as broad umbrella terms in the title, whereas psychological regulation processes (ACSI-28) and mental performance expression (SMTQ) represent their operationalized constructs in the present study.

### Trait mindfulness (MAAS)

Trait mindfulness was measured using the 15-item Mindful Attention Awareness Scale (MAAS). The scale assesses the dispositional tendency to attend to present-moment experiences with awareness. Items are rated on a 6-point Likert scale (1 = almost always, 6 = almost never), with higher scores indicating higher mindfulness. Previous studies support its reliability and validity in athlete populations [39]. The mean score across all items was used in analyses. The Chinese version of MAAS has been previously validated in Chinese populations and demonstrated good psychometric properties [40].

### Psychological regulation processes (ACSI-28)

Psychological regulation processes were assessed using the Athletic Coping Skills Inventory-28 (ACSI-28) [41], consisting of seven subscales: coping with adversity, peaking under pressure, goal setting or mental preparation, focus, freedom from worry, confidence and achievement motivation, and coachability. Items 17, 18, 19, 20, 26, and 28 were reverse scored. Responses use a 4-point Likert scale (0 = almost never, 3 = almost always). The Chinese version of ACSI-28 has been validated in Chinese athlete populations, demonstrating acceptable reliability and construct validity [42].

In the present study, the total ACSI-28 score was used for all inferential analyses. Subscale scores were excluded from hypothesis testing due to low internal consistency (α = 0.32–0.37) and were treated as exploratory only.

### Mental performance expression (SMTQ)

Mental performance expression was measured using the Sport Mental Toughness Questionnaire (SMTQ) [43], comprising three subscales: confidence, constancy, and control. Items 9–14 were reverse scored. Responses used a 4-point Likert scale (1 = strongly disagree, 4 = strongly agree). Cronbach’s α for the total scale was 0.87. The SMTQ is widely used in sport psychology, although conceptual and measurement issues remain. A validated Chinese version has been previously reported in sport-specific contexts, supporting its applicability in athletic populations [44].

### Data collection procedure

All participants completed the questionnaires in an indoor classroom near the training base. Standardized instructions were provided, and researchers demonstrated how to complete the Likert scales. Measures were completed in the following order: Demographics, MAAS, ACSI-28, SMTQ. To avoid fatigue-related bias, testing occurred before formal training on the same day, with total testing time approximately 20 minutes.

### Statistical analysis

Analyses were conducted using SPSS 27.0 (IBM Corp., Armonk, NY, USA). Descriptive statistics were used to summarize demographic and study variables. Categorical variables were reported as frequencies and percentages, whereas continuous variables were expressed as means, standard deviations, and ranges. Normality was assessed using skewness and kurtosis values within ±2.

Statistical power was considered based on G*Power 3.1 [45], indicating that the present sample size (N = 172) is sufficient to achieve adequate power (≥ 0.80) for detecting medium effect sizes in regression-based models. Internal consistency was assessed using Cronbach’s alpha. Independent-samples t-tests were used to examine gender differences, with Levene’s test applied to assess homogeneity of variance. Effect sizes were reported using Cohen’s d and 95% confidence intervals (CI).

Pearson correlation analyses were conducted to examine associations among trait mindfulness, psychological regulation processes, and mental performance expression. False discovery rate (FDR) correction was applied to control for multiple comparisons. Mediation analysis was performed using 5,000 bootstrap resamples, with the total score of psychological regulation processes (ACSI-28) specified as the mediating variable between trait mindfulness and mental performance expression.

Regression assumptions were evaluated using the Shapiro–Wilk test, Breusch–Pagan test, Durbin–Watson statistic, variance inflation factor (VIF), and Cook’s distance [46]. Measurement model quality was assessed using composite reliability (CR) and average variance extracted (AVE) for ACSI-28 and SMTQ, and discriminant validity was evaluated using the Fornell–Larcker criterion.

Abbreviations used in the present study include confidence interval (CI), false discovery rate (FDR), variance inflation factor (VIF), composite reliability (CR), average variance extracted (AVE), comparative fit index (CFI), Tucker–Lewis index (TLI), root mean square error of approximation (RMSEA), and standardized root mean square residual (SRMR). Cross-sectional design limits interpretation to statistical associations and does not allow inference of causal relationships.

## Results

### Participant characteristics and preliminary analyses

A total of 172 competitive paddleboard athletes were included in the study, consisting of 87 males and 85 females. The mean age was 23.99 years (SD = 3.55, range 18–30). Athletes had between 1 and 10 years of paddleboard training experience (M = 4.54, SD = 1.87), and their mean daily training duration was 3.21 hours (SD = 0.69, range 1.5–4.8). Participant characteristics are summarized in Table 1, for descriptive purposes.

**Table 1:** Participant characteristics of competitive paddleboard athletes.

Panel A. Categorical characteristics			
**Variable**	**Category**	**n**	**%**
Gender	Male	87	50.6
	Female	85	49.4
Age group	18-21 years	46	26.7
	22-25 years	71	41.3
	26-30 years	55	32.0
Athletic level	International elite athlete	3	1.7
	National elite athlete	14	8.1
	Level 1	46	26.7
	Level 2	65	37.8
	None	44	25.6
Paddleboard event	Four-person paddleboard	36	20.9
	Double paddleboard	57	33.1
	Single paddleboard	79	45.9
Training experience group	1-3 years	40	23.3
	4-6 years	100	58.1
	>6 years	32	18.6
Panel B. Continuous characteristics			
**Variable**	**M**	**SD**	**Range**
Age (years)	23.99	3.55	18-30
Daily training duration (hours)	3.21	0.69	1.5-4.8
Total years of paddleboard training	4.54	1.87	1-10

**Note.** Total sample size was N = 172. Panel A presents categorical variables as frequencies (percentages), and Panel B presents continuous variables as mean ± standard deviation and range.

### Descriptive statistics, normality, and reliability of study variables

Descriptive statistics, normality indices, and internal consistency coefficients are presented in Table 2. Athletes reported moderate-to-high levels of trait mindfulness, psychological regulation processes, and mental performance expression. Skewness and kurtosis values were all within the acceptable range (±2), indicating acceptable distributional properties for parametric analyses.

**Table 2:** Descriptive statistics, normality, and reliability within the study variables.

Measure	N	M	SD	Min	Max	Skewness	Kurtosis	Cronbach’s α
Trait mindfulness (MAAS)	172	4.23	0.61	2.00	5.73	−0.36	0.42	.88
Psychological regulation processes (ACSI-28 total)	172	51.54	6.59	30.00	67.00	−0.25	0.40	.87
Focus	172	7.65	1.21	3.00	12.00	−0.05	1.55	.37
Coping with adversity	172	7.65	1.12	4.00	11.00	−0.32	0.47	.32
Peaking under pressure	172	7.35	1.12	4.00	10.00	−0.17	−0.16	.33
Mental performance expression (SMTQ total)	172	38.45	5.19	20.00	51.00	−0.21	0.18	.87
Confidence	172	10.93	1.71	5.00	15.00	−0.15	0.40	.52
Constancy	172	11.77	1.70	7.00	16.00	0.08	−0.16	.64
Control	172	15.75	2.14	8.00	20.00	−0.33	0.25	.69

**Note.** N = 172. Cronbach’s α is reported for total scales and selected subscales. Total scores were used for primary analyses in the present analysis, while subscales were treated as exploratory due to low internal consistency.

Internal consistency was acceptable for all total scales (MAAS α = 0.88, ACSI-28 total α = 0.87, SMTQ α = 0.87). All inferential analyses in this study were conducted using total scale scores only. Subscale-level variables were not included in CFA, correlation, regression, or any structural analyses due to low internal consistency and were removed from inferential modeling.

### Gender differences

No significant gender differences were observed in trait mindfulness, psychological regulation processes, or mental performance expression (Table 3). Effect sizes were trivial to small across all variables, indicating no statistically significant subgroup differences. These results support pooling male and female participants for subsequent correlational analyses, indicating stability of the study variables across gender groups.

**Table 3:** Gender differences in study variables variables.

Variable	Male n	Male M ± SD	Female n	Female M ± SD	Levene p	t	df	p	Mean difference	95% CI for difference	Cohen’s d
Trait mindfulness (MAAS)	87	4.25 ± 0.58	85	4.20 ± 0.64	.176	0.52	170	.601	0.05	[-0.14, 0.23]	0.08
psychological regulation processess (ACSI-28 total)	87	51.37 ± 6.53	85	51.72 ± 6.68	.839	−0.35	170	.729	−0.35	[-2.34, 1.64]	−0.05
mental performance expression (SMTQ total)	87	38.55 ± 4.81	85	38.34 ± 5.59	.340	0.27	170	.791	0.21	[-1.36, 1.78]	0.04

**Note.** Mean difference = male minus female. Cohen’s d was calculated using pooled standard deviation. Levene’s test assessed homogeneity of variance. Psychological regulation processes and mental performance expression represent study variables. All p values > .05 indicated no statistically significant gender differences. Effect sizes were trivial, supporting practical invariance across gender groups. Therefore, data were pooled for subsequent analyses.

### Correlations among main variables

Pearson correlation analyses were conducted to examine associations among trait mindfulness and components of study variables, including psychological regulation processes and mental performance expression. All analyses were based exclusively on total scale scores. No subscale-level variables were included in inferential analyses. No subscale-level variables were included in inferential analyses.

Trait mindfulness was positively associated with psychological regulation processes (r = 0.642, 95% CI [0.545, 0.722]) and mental performance expression (r = 0.498, 95% CI [0.376, 0.603]).

Psychological regulation processes showed the highest correlation with mental performance expression (r = 0.772, 95% CI [0.704, 0.826]) Table 4.

**Table 4:** Statistical correlations among psychological functioning components.

Hypothesis	Association	r	95% CI	p	FDR-adjusted p	r²	Effect size
H1	Trait mindfulness-psychological regulation processes	.642	[.545,.722]	<.001	<.001	.412	Large
H2	Trait mindfulness-mental performance expression	.498	[.376,.603]	<.001	<.001	.248	Medium
H3	psychological regulation processes-mental performance expression	.772	[.704,.826]	<.001	<.001	.596	Large

**Note.** MAAS = Mindful Attention Awareness Scale; ACSI-28 = Athletic Coping Skills Inventory-28 (psychological regulation processes); SMTQ = Sport Mental Toughness Questionnaire (mental performance expression); CI = confidence interval; FDR = false discovery rate. Effect sizes were interpreted according to Cohen’s criteria. Subscale-level correlations were exploratory due to low internal consistency of selected ACSI-28 subscales. All variables are treated as observed measures in this study.

### Subscale-level correlations

The subcomponents of psychological regulation processes (Focus, Coping with adversity, and Peaking under pressure) were positively associated with the dimensions of mental performance expression (Confidence, Constancy, and Control) (Table 5).

**Table 5:** Statistical subcomponent associations.

Variable	Confidence	Constancy	Control
Trait mindfulness	.467 [.341,.576]	.429 [.298,.544]	.492 [.370,.598]
Focus (psychological regulation subcomponent)	.602 [.497,.689]	.562 [.450,.656]	.589 [.482,.679]
Coping with adversity	.648 [.551,.727]	.599 [.493,.687]	.643 [.546,.723]
Peaking under pressure	.616 [.514,.701]	.535 [.419,.634]	.606 [.501,.692]

Note. Values are Pearson correlation coefficients with 95% confidence intervals. Trait mindfulness was measured using MAAS. Focus, Coping with adversity, and Peaking under pressure represent subcomponents of psychological regulation processes. Confidence, Constancy, and Control represent dimensions of mental performance expression. All correlations were statistically significant at p < 0.001 and remained significant after FDR correction. These analyses reflect within-system functional associations. Subscale-level results should be interpreted cautiously due to low internal consistency at the subcomponent level.

The strongest correlations were observed between Coping with adversity and Confidence (r = 0.648, 95% CI [0.551, 0.727]) and between Coping with adversity and Control (r = 0.643, 95% CI [0.546, 0.723]).

### Statistical pathway analysis

The ACSI-28 total score was examined as a statistical association variable in the relationships among trait mindfulness, psychological regulation processes, and mental performance expression (Table 6). Trait mindfulness showed significant statistical associations with both psychological regulation processes and mental performance expression. Psychological regulation processes were also strongly associated with mental performance expression within the present sample.

**Table 6:** Statistical association parameters among study variables within a cross-sectional covariance model.

Path	Relationship	b	SE	β	t	p	95% CI
a	Trait mindfulness →Psychological regulation processes	6.920	0.634	.642	10.91	<.001	[5.669, 8.172]
b	Psychological regulation processes →Mental performance expression	0.607	0.050	.769	12.07	<.001	[0.507, 0.706]
c	Trait mindfulness → Mental performance expression	4.232	0.565	.498	7.49	<.001	[3.116, 5.348]
c′	Trait mindfulness → Mental performance expression controlling for psychological skills	0.035	0.542	.004	0.06	.949	[-1.035, 1.105]
a × b	Combined association term (a × b) describing covariance structure among variables	4.197	—	—	—	—	[3.165, 5.340]

**Note.** All variables were analyzed within a cross-sectional covariance framework. Path coefficients represent statistical associations among variables rather than causal relationships. The combined term (a × b) is reported to describe the covariance structure among variables within the model. No causal mediation is inferred in this analysis.

The inclusion of psychological regulation processes did not alter the interpretation of these relationships as statistical associations. The observed pattern reflects shared variance among variables within a cross-sectional covariance framework rather than directional or causal effects.

### Assumption checks and construct-overlap, and Common Method Bias

Regression assumptions were examined prior to hypothesis testing. The results indicated that normality, homoscedasticity, and independence assumptions were all satisfied. Multicollinearity diagnostics further showed acceptable variance inflation factor (VIF) and tolerance values, suggesting no multicollinearity concerns in the regression model (Table 7).

**Table 7:** Regression assumptions and multicollinearity diagnostics.

Test	Result
Shapiro–Wilk	.741
Breusch–Pagan	.759
Durbin-Watson	1.89
VIF(max)	1.701
Tolerance (min)	0.588

**Note.** Shapiro–Wilk test was used to assess normality; Breusch–Pagan test was used to assess homoscedasticity; Durbin–Watson statistic was used to assess independence of residuals. VIF = variance inflation factor; tolerance values were used to assess multicollinearity. All assumptions were evaluated at the α = .05 significance level.

To further examine the interrelationship between psychological regulation processes and mental performance expression, correlation-based analyses were conducted. Results indicated a strong association between the two constructs (r = 0.772, r² = 0.596), reflecting substantial shared variance within the self-regulation system examined in this study.

Given that all variables were collected using self-report questionnaires at a single time point, common method bias was assessed using Harman’s single-factor test. The results showed that the first factor accounted for 27.42% of the total variance, which is below the recommended threshold of 40%, indicating that common method variance is unlikely to be a serious concern in the present study.

### Dimension-based diagnostics and CFA

**Fig 2:**
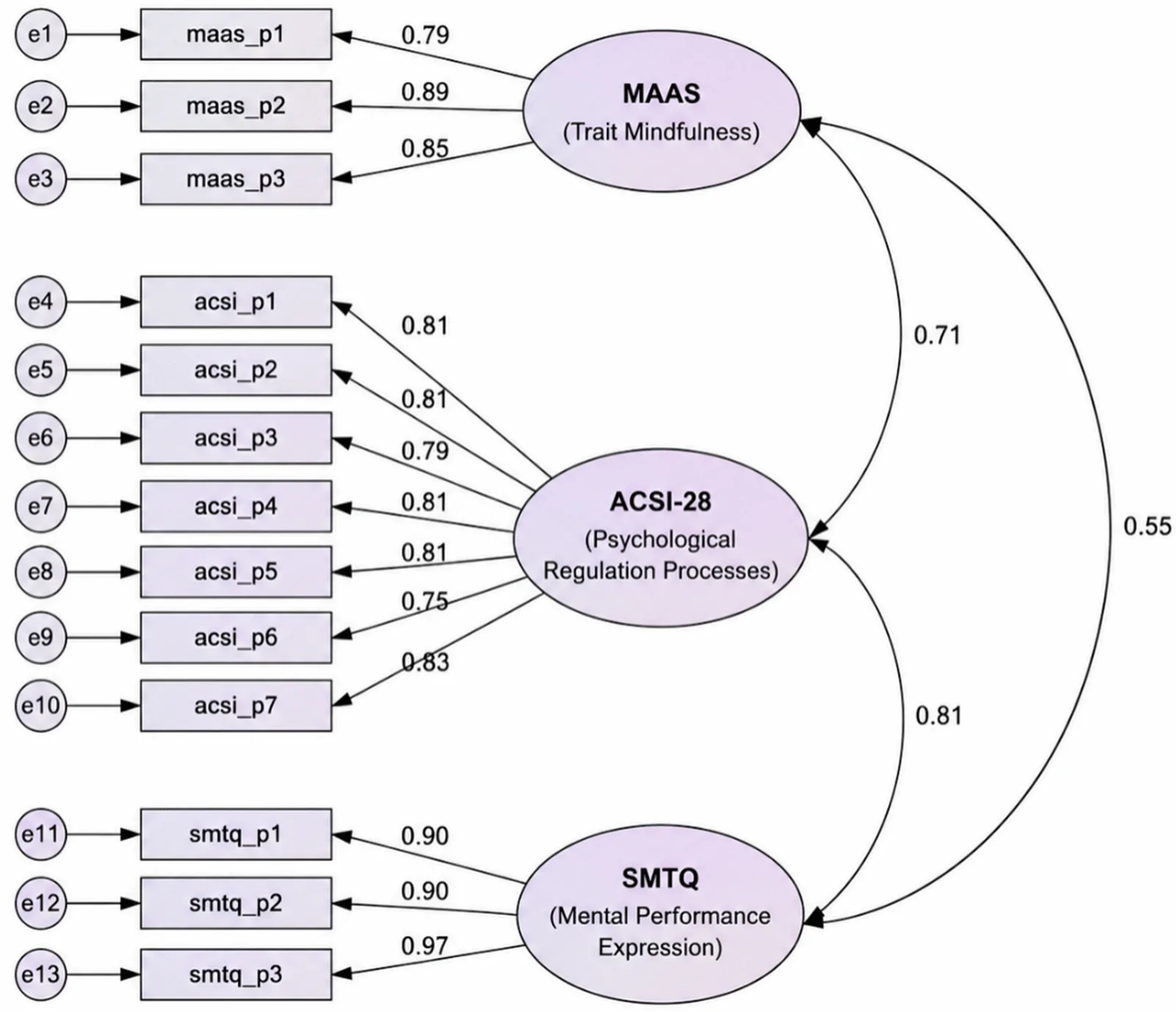
CFA measurement model of the three latent constructs in paddleboard athletes. **Note.** The results indicated an acceptable to good model fit (CFI and TLI > 0.95; RMSEA and SRMR < 0.08), with CFI and TLI indicating excellent incremental fit and RMSEA and SRMR indicating acceptable absolute fit.

Composite reliability (CR) and average variance extracted (AVE) are reported in Table 8. All inferential analyses were conducted using total scale scores only Table 9. Subscale-level indicators were excluded from CFA, SEM, and hypothesis testing due to low internal consistency. Confirmatory factor analysis results are reported in Table 10. The measurement model demonstrated excellent overall fit (,x2/df = 1.45, CFI = 0.958, TLI = 0.965, RMSEA = 0.067,SRMR = 0.065, GFI = 0.951) Fig 2 and Table 11.

**Table 10:** Measurement adequacy within the study variables.

Construct	Indicators used	CR	AVE	√AVE
Trait mindfulness	3 parcels	.881	.712	.844
Psychological regulation processes	7 subscales	.926	.641	.801
Mental performance expression	3 dimensions	.964	.899	.948

**Note.** Parcel-level indicators were used for confirmatory factor analysis due to improved reliability and reduced measurement error. CR = composite reliability; AVE = average variance extracted.

**Table 11:** Confirmatory Factor Analysis Model Fit.

Variable	χ²/df	CFI	GFI	TLI	RMSEA	SRMR
Three-factor measurement model	1.45	0.958	0.951	0.965	0.067	0.065

**Note.** The measurement model was evaluated using confirmatory factor analysis (CFA) with maximum likelihood estimation in AMOS. Model fit was assessed using multiple indices, including χ²/df, CFI, TLI, RMSEA, SRMR, and GFI. The results indicated an acceptable to good model fit (CFI and TLI > 0.95; RMSEA and SRMR < 0.08), supporting the adequacy of the proposed three-factor measurement structure. A parceling approach was adopted to improve model parsimony and reduce random measurement error. The CFA was conducted at the parcel level.

**Table 8:** Construct overlap between psychological regulation processes and mental performance expression.

Relationship	r	r²
Psychological regulation processes – Mental performance expression	.772	.596

Note. Values represent Pearson correlation coefficient and shared variance (r²). This analysis was used to describe the covariance structure between variables within a self-regulation framework.

**Table 9:** Harman’s single-factor test.

Metric	Value
First factor variance explained	27.425
Total variance	100%

**Note.** Common method bias was assessed using Harman’s single-factor test. The percentage of variance explained by the first unrotated factor was used as an indicator of potential common method variance.

## Discussion

The present study examined the measurement structure and statistical associations among trait mindfulness, psychological regulation processes, and mental performance expression in competitive paddleboard athletes. Findings were interpreted within a cross-sectional covariance framework, emphasizing statistical associations rather than causal relationships.

Participants exhibited moderate-to-high levels across all measured constructs, reflecting the cognitive and regulatory demands of paddleboarding, including sustained attentional control, continuous environmental adaptation, and performance stability under unstable aquatic conditions. Internal consistency was acceptable at the construct level for MAAS, ACSI-28 total, and SMTQ. However, certain ACSI-28 subcomponents demonstrated low reliability and were therefore treated as exploratory indicators.

The confirmatory factor analysis indicated an acceptable three-factor measurement structure with strong global fit indices. This supports that trait mindfulness, psychological regulation processes, and mental performance expression can be statistically represented as distinct but correlated constructs within the present dataset. However, inter-construct correlations indicated substantial shared variance, particularly between psychological regulation processes and mental performance expression.

Importantly, the relatively high correlation between psychological regulation processes and mental performance expression (r = 0.772) does not indicate measurement redundancy. Instead, this pattern reflects an interconnected but empirically distinguishable pattern of psychological constructs, consistent with functional coupling within sport-specific self-regulation processes.

From this perspective, discriminant validity is interpreted as partial rather than absolute, reflecting theoretical proximity and functional interdependence rather than construct redundancy. The observed pattern is therefore better understood as system-level covariance within a shared regulatory context.

Gender differences were not statistically significant across all variables, with small effect sizes and confidence intervals indicating no reliable group differences in this sample.

From a practical perspective, the present findings suggest that trait mindfulness and psychological regulation processes may represent relevant psychological targets in paddleboarding training contexts. The associations observed indicate that athletes with higher mindfulness tend to report stronger regulatory capacities, which are associated with strongerperformance-related psychological profiles [47]. Although causal interpretations cannot be made due to the cross-sectional design, these results may provide a basis for further evaluating psychological skills training programs focusing on attentional control, emotional regulation, and coping strategies in balance-demanding aquatic sports [48].

At the subcomponent level, exploratory analyses indicated associations between trait mindfulness, psychological regulation components, and dimensions of mental performance expression. However, these findings should be interpreted cautiously due to reduced reliability of several ACSI-28 subscales.

Several limitations should be acknowledged. The cross-sectional design precludes causal inference, and self-report measures may introduce shared method variance. In addition, inter-construct correlations likely reflect both conceptual proximity and functional integration within sport-specific psychological processes. Future longitudinal and experimental research is required to clarify temporal and directional relationships among these variables.

## Conclusions

This study examined trait mindfulness, psychological regulation processes, and mental performance expression in competitive paddleboard athletes. Participants demonstrated moderate-to-high levels across psychological variables, with no significant gender differences observed.

Trait mindfulness showed significant statistical associations with both psychological regulation processes and mental performance expression. Psychological regulation processes were also strongly associated with mental performance expression within the present sample.

The observed pattern of associations suggests that psychological regulation processes are statistically associated with the relationship between trait mindfulness and mental performance expression in this cross-sectional dataset.

Overall, the findings contribute to sport psychology literature by highlighting an interconnected pattern of statistical associations among mindfulness, regulatory processes, and performance-related variables in a balance-demanding sport context such as paddleboarding.

### Limitations

The present study has several limitations. First, as all variables were collected using self-report questionnaires at a single time point, the findings may be subject to common source bias (common method variance), which may inflate observed associations among variables. Although statistical checks were conducted, including Harman’s single-factor test, the possibility of residual method bias cannot be entirely ruled out.

Second, the use of self-report measures may introduce response bias, including social desirability bias and subjective over- or under-reporting of psychological states and performance-related characteristics.

Third, the relatively high correlation observed between psychological regulation processes and mental performance expression (r = .772) suggests substantial shared variance between the constructs, which may reflect conceptual proximity and functional interdependence within a broader self-regulation framework rather than strict discriminant separability.

Fourth, several ACSI-28 subscales demonstrated low internal consistency (Focus α = .37; Coping with Adversity α = .32; Peaking Under Pressure α = .33), indicating that subscale-level results should be interpreted with caution and considered exploratory.

Finally, although mediation analysis was conducted within a statistical framework, the cross-sectional design precludes establishing temporal precedence. Therefore, the observed indirect effect should be interpreted strictly as a statistical association pattern rather than evidence of a causal mediating mechanism.

Future research should adopt multi-method designs and longitudinal approaches to reduce potential bias and strengthen inference validity.

## References

[1] Swann C, Moran A, Piggott D. Defining elite athletes: issues in the study of expert performance in sport psychology. Psychology of Sport and Exercise. 2015;16:3–14. doi: 10.1016/j.psychsport.2014.07.004

[2] Brown KW, Ryan RM. The benefits of being present: mindfulness and its role in psychological well-being. J Pers Soc Psychol. 2003;84(4):822–48. doi: 10.1037/0022-3514.84.4.822 12703651

[3] Smith RE, Schutz RW, Smoll FL, Ptacek JT. Development and validation of a multidimensional measure of sport-specific psychological skills: the Athletic Coping Skills Inventory-28. Journal of Sport and Exercise Psychology. 1995;17(4):379–98. doi: 10.1123/jsep.17.4.379

[4] Sheard M, Golby J, van Wersch A. Progress Toward construct validation of the sports mental toughness questionnaire (SMTQ). European Journal of Psychological Assessment. 2009;25(3):186–93. doi: 10.1027/1015-5759.25.3.186

[5] Wu C-H, Nien J-T, Lin C-Y, Nien Y-H, Kuan G, Wu T-Y, et al. Relationship between mindfulness, psychological skills, and mental toughness in college athletes. Int J Environ Res Public Health. 2021;18(13):6802. doi: 10.3390/ijerph18136802 34202770 PMC8297292

[6] Blakely MJ, Smith SL, Russell PN, Helton WS. The impact of cognitive load on kayaking and kayaking on cognitive performance. Appl Ergon. 2022;102:103747. doi: 10.1016/j.apergo.2022.103747 35306246

[7] Prétot C, Carmigniani R, Hasbroucq L, Labbé R, Boucher J-P, Clanet C. On the physics of kayaking. Applied Sciences. 2022;12(18):8925. doi: 10.3390/app12188925

[8] Gäbler M, Berberyan HS, Prieske O, Elferink-Gemser MT, Hortobágyi T, Warnke T, et al. Strength training intensity and volume affect performance of young kayakers/canoeists. Front Physiol. 2021;12:686744. doi: 10.3389/fphys.2021.686744 34248673 PMC8264585

[9] Cowden RG. Mental toughness and success in sport: a review and prospect. TOSSJ. 2017;10(1):1–14. doi: 10.2174/1875399x01710010001

[10] Michael JS, Rooney KB, Smith R. The metabolic demands of kayaking: a review. J Sports Sci Med. 2008;7(1):1–7. 24150127 PMC3763332

[11] Liew GC, Kuan G, Chin NS, Hashim HA. Mental toughness in sport. Ger J Exerc Sport Res. 2019;49(4):381–94. doi: 10.1007/s12662-019-00603-3

[12] Hsieh Y-C, Lu FJH, Gill DL, Hsu Y-W, Wong T-L, Kuan G. Effects of mental toughness on athletic performance: a systematic review and meta-analysis. International Journal of Sport and Exercise Psychology. 2023;22(6):1317–38. doi: 10.1080/1612197x.2023.2204312

[13] Fletcher D, Sarkar M. A grounded theory of psychological resilience in Olympic champions. Psychology of Sport and Exercise. 2012;13(5):669–78. doi: 10.1016/j.psychsport.2012.04.007

[14] Zhong Z, Jiang H, Wang H, Liu Y. Mindfulness, social evaluation anxiety, and self-regulation: exploring their association on impulsive behavior among athletes. Front Psychiatry. 2024;15:1404680. doi: 10.3389/fpsyt.2024.1404680 38807692 PMC11130505

[15] Rogowska AM, Tataruch R. The relationship between mindfulness and athletes’ mental skills may be explained by emotion regulation and self-regulation. BMC Sports Sci Med Rehabil. 2024;16(1):68. doi: 10.1186/s13102-024-00863-z 38504372 PMC10949773

[16] Bagheri S, Naderi A, Mirali S, Calmeiro L, Brewer BW. Adding mindfulness practice to exercise therapy for female recreational runners with patellofemoral pain: a randomized controlled trial. J Athl Train. 2021;56(8):902–11. doi: 10.4085/1062-6050-0214.20 33237990 PMC8359715

[17] Koop L, Jooste J. Mindfulness traits as potential inhibitor of irrational performance beliefs and intolerance of uncertainty amongst elite female basketball players. Apunts Sports Medicine. 2023;58(220):100428. doi: 10.1016/j.apunsm.2023.100428

[18] Röthlin P, Horvath S, Trösch S, Holtforth MG, Birrer D. Differential and shared effects of psychological skills training and mindfulness training on performance-relevant psychological factors in sport: a randomized controlled trial. BMC Psychol. 2020;8(1):80. doi: 10.1186/s40359-020-00449-7 32762736 PMC7409666

[19] Gucciardi DF, Hanton S, Gordon S, Mallett CJ, Temby P. The concept of mental toughness: tests of dimensionality, nomological network, and traitness. J Pers. 2015;83(1):26–44. doi: 10.1111/jopy.12079 24428736

[20] Goddard K, Roberts C-M, Anderson L, Woodford L, Byron-Daniel J. Mental toughness and associated personality characteristics of marathon des sables athletes. Front Psychol. 2019;10:2259. doi: 10.3389/fpsyg.2019.02259 31636592 PMC6787285

[21] Josefsson T, Ivarsson A, Lindwall M, Gustafsson H, Stenling A, Böröy J, et al. Mindfulness mechanisms in sports: mediating effects of rumination and emotion regulation on sport-specific coping. Mindfulness (N Y). 2017;8(5):1354–63. doi: 10.1007/s12671-017-0711-4 28989551 PMC5605575

[22] Si XW, Yang ZK, Feng X. A meta-analysis of the intervention effect of mindfulness training on athletes’ performance. Front Psychol. 2024;15:1375608. doi: 10.3389/fpsyg.2024.1375608 38939219 PMC11210447

[23] Crust L. A review and conceptual re-examination of mental toughness: implications for future researchers. Personality and Individual Differences. 2008;45(7):576–83. doi: 10.1016/j.paid.2008.07.005

[24] Nicholls AR, Polman RCJ, Levy AR, Backhouse SH. Mental toughness in sport: achievement level, gender, age, experience, and sport type differences. Personality and Individual Differences. 2009;47(1):73–5. doi: 10.1016/j.paid.2009.02.006

[25] Soundara Pandian PR, Balaji Kumar V, Kannan M, Gurusamy G, Lakshmi B. Impact of mental toughness on athlete’s performance and interventions to improve. J Basic Clin Physiol Pharmacol. 2022;34(4):409–18. doi: 10.1515/jbcpp-2022-0129 35792085

[26] Naderi A, Shaabani F, Gharayagh Zandi H, Calmeiro L, Brewer BW. The effects of a mindfulness-based program on the incidence of injuries in young male soccer players. J Sport Exerc Psychol. 2020;42(2):161–71. doi: 10.1123/jsep.2019-0003 32150722

[27] Gardner FL, Moore ZE. Mindfulness and acceptance models in sport psychology: a decade of basic and applied scientific advancements. Canadian Psychology / Psychologie canadienne. 2012;53(4):309–18. doi: 10.1037/a0030220

[28] Gucciardi DF. Mental toughness: progress and prospects. Curr Opin Psychol. 2017;16:17–23. doi: 10.1016/j.copsyc.2017.03.010 28813344

[29] Crust L. Mental toughness in sport: a review. International Journal of Sport and Exercise Psychology. 2007;5(3):270–90. doi: 10.1080/1612197x.2007.9671836

[30] Connaughton D, Wadey R, Hanton S, Jones G. The development and maintenance of mental toughness: perceptions of elite performers. J Sports Sci. 2008;26(1):83–95. doi: 10.1080/02640410701310958 17852671

[31] Wang Y, Lei S-M, Fan J. Effects of mindfulness-based interventions on promoting athletic performance and related factors among athletes: a systematic review and meta-analysis of randomized controlled trial. Int J Environ Res Public Health. 2023;20(3):2038. doi: 10.3390/ijerph20032038 36767403 PMC9915077

[32] Birrer D, Morgan G. Psychological skills training as a way to enhance an athlete’s performance in high-intensity sports. Scand J Med Sci Sports. 2010;20 Suppl 2:78–87. doi: 10.1111/j.1600-0838.2010.01188.x 20840565

[33] Bull SJ, Shambrook CJ, James W, Brooks JE. Towards an understanding of mental toughness in elite english cricketers. Journal of Applied Sport Psychology. 2005;17(3):209–27. doi: 10.1080/10413200591010085

[34] Shaabani F, Naderi A, Borella E, Calmeiro L. Does a brief mindfulness intervention counteract the detrimental effects of ego depletion in basketball free throw under pressure?. Sport, Exercise, and Performance Psychology. 2020;9(2):197–215. doi: 10.1037/spy0000201

[35] Jones MI, Parker JK. Mindfulness mediates the relationship between mental toughness and pain catastrophizing in cyclists. Eur J Sport Sci. 2018;18(6):872–81. doi: 10.1080/17461391.2018.1478450 29870312

[36] Thomas JR, Nelson JK, Silverman SJ. Research Methods in Physical Activity. 7th ed. Champaign: Human Kinetics. 2015.

[37] World Medical Association. World Medical Association Declaration of Helsinki: ethical principles for medical research involving human subjects. JAMA. 2013;310(20):2191–4. doi: 10.1001/jama.2013.281053 24141714

[38] Naderi A, Alizadeh N, Calmeiro L, Degens H. Predictors of running-related injury among recreational runners: a prospective cohort study of the role of perfectionism, mental toughness, and passion in running. Sports Health. 2024;16(6):1038–49. doi: 10.1177/19417381231223475 38311884 PMC11531021

[39] Josefsson T, Ivarsson A, Lindwall M, Gustafsson H, Stenling A, Böröy J, et al. Mindfulness mechanisms in sports: mediating effects of rumination and emotion regulation on sport-specific coping. Mindfulness (N Y). 2017;8(5):1354–63. doi: 10.1007/s12671-017-0711-4 28989551 PMC5605575

[40] Deng Y-Q, Li S, Tang Y-Y, Zhu L-H, Ryan R, Brown K. Psychometric properties of the Chinese translation of the mindful attention awareness scale (MAAS). Mindfulness. 2011;3(1):10–4. doi: 10.1007/s12671-011-0074-1

[41] Sheard M, Golby J. Effect of a psychological skills training program on swimming performance and positive psychological development. International Journal of Sport and Exercise Psychology. 2006;4(2):149–69. doi: 10.1080/1612197x.2006.9671790

[42] Yan Z, Mok MMC. Validating the coping scale for Chinese athletes using multidimensional Rasch analysis. Psychology of Sport and Exercise. 2012;13(3):271–9. doi: 10.1016/j.psychsport.2011.11.013

[43] Cohen J. A power primer. Psychol Bull. 1992;112(1):155–9. doi: 10.1037/0033-2909.112.1.155 19565683

[44] Partikova V. Testing the mental toughness model in the setting of chinese martial arts. ajper. 2018;24(1):36–43. doi: 10.24112/ajper.241759

[45] Faul F, Erdfelder E, Buchner A, Lang A-G. Statistical power analyses using G*Power 3.1: tests for correlation and regression analyses. Behav Res Methods. 2009;41(4):1149–60. doi: 10.3758/BRM.41.4.1149 19897823

[46] Field A. Discovering Statistics Using IBM SPSS Statistics. 5th ed. London: Sage. 2018.

[47] Zhong Z, Jiang H, Wang H, Liu Y. The association between mindfulness and athletes’ distress tolerance: the mediating roles of cognitive reappraisal and mental toughness. Behav Sci (Basel). 2025;15(3):298. doi: 10.3390/bs15030298 40150193 PMC11939238

[48] Reinebo G, Alfonsson S, Jansson-Fröjmark M, Rozental A, Lundgren T. Effects of psychological interventions to enhance athletic performance: a systematic review and meta-analysis. Sports Med. 2024;54(2):347–73. doi: 10.1007/s40279-023-01931-z 37812334 PMC10933186

